# Anticipatory and compensatory postural adjustments in people with low back pain: a protocol for a systematic review and meta-analysis

**DOI:** 10.1186/s13643-016-0242-4

**Published:** 2016-04-16

**Authors:** Michael F. Knox, Lucy S. Chipchase, Siobhan M. Schabrun, Paul W. M. Marshall

**Affiliations:** School of Science and Health, Western Sydney University, Campbelltown Campus, Room 20.G.35, Locked bag 1797, Penrith, 2751 New South Wales Australia

**Keywords:** Low back pain, Anticipatory, Compensatory, Postural adjustment, Postural control, Systematic review

## Abstract

**Background:**

Anticipatory (APAs) and compensatory (CPAs) postural adjustments are organised by the central nervous system (CNS) and serve to control postural perturbations. Ineffective APAs and CPAs have been hypothesised to contribute to the persistence of symptoms and disability in people with low back pain (LBP). Despite two decades of research, there is no systematic review investigating APAs and CPAs in people with LBP. Thus, the aim of the current review is to determine if APA and CPA onset or amplitude, as measured by electromyography (EMG), centre of pressure (COP), and kinematics, are altered in people with LBP.

**Methods/design:**

A systematic review and meta-analysis will be conducted. Searches will be conducted in electronic databases for full-text articles published before January 2016 using pre-defined search strategies that utilise combinations of keywords and medical subject heading terms. Two independent reviewers will screen potentially relevant articles for inclusion, extract data, and assess risk of bias for individual studies. Any disagreements will be resolved by a third reviewer. Studies comparing APA onset and amplitude and CPA onset and amplitude measured by EMG, COP, or kinematics between people with LBP and healthy individuals will be included if all aspects of the eligibility criteria are met. Data will be synthesised if studies are homogeneous; otherwise, results will be reviewed narratively.

**Discussion:**

To our knowledge, this is the first systematic review to examine APAs and CPAs, as measured by EMG, COP, and kinematics in people with LBP. The findings of this review may aid in the identification of factors that play a role in the persistence of symptoms and disability and aid in the development of interventions to treat symptoms.

**Systematic review registration:**

PROSPERO CRD42016032815

**Electronic supplementary material:**

The online version of this article (doi:10.1186/s13643-016-0242-4) contains supplementary material, which is available to authorized users.

## Background

Low back pain (LBP) is a leading cause of work absence and activity limitation worldwide [1]. Approximately 84 % of people will experience an acute episode of LBP [2], with estimates that 10 % of these will develop chronic symptoms [3]. Despite the high prevalence, the effect sizes of most treatments are small [4]. The identification of biological factors that play a role in the persistence of symptoms is vital for the development of interventions to treat LBP.

A plausible biological mechanism that may impede LBP recovery is the control of postural perturbations by the central nervous system (CNS). Authors hypothesise that ineffective control of postural perturbations increases the risk of excessive forces being experienced by passive structures of the spine [5, 6], contributing to the persistence of LBP. During activities of daily living, people may experience postural perturbations in the form of self-initiated movements, standing on a moving bus, or being hit by an external object. Anticipatory (APAs) and compensatory (CPAs) postural adjustments are alterations in muscle activity by the CNS to control forces imparted on the body by postural perturbations [7]. APAs can be observed prior to a predictable perturbation and serve to minimise the perturbation’s effect [8–10], while CPAs are observed following a perturbation and are a mechanism to re-establish postural equilibrium [11, 12]. Studies have associated cortical structures with the planning and generation of APAs [13–15], while CPAs integrate sensory feedback responses and voluntary commands [7, 16, 17]. Identifying altered APAs and CPAs in the presence of LBP may aid in the identification of structural changes within the CNS and subsequently assist with determining optimal interventions for LBP.

Studies examining postural adjustments between people with LBP and healthy individuals have used electromyography (EMG) [18, 19], centre of pressure (COP) [20, 21], and kinematics [22, 23]. Two systematic reviews have examined differences in the control of standing posture, measured by COP only, in people with LBP and healthy individuals [23, 24]. These reviews provide evidence of changes in the control of standing posture in the presence of LBP. However, these reviews excluded studies utilising postural perturbations and therefore do not provide insight into changes in APAs and CPAs in the presence of LBP. Furthermore, no published systematic review has examined if APAs and CPAs are altered in the presence of LBP. Thus, the aim of this systematic review is to determine if APA or CPA onset or amplitude, as measured by EMG, COP, and kinematics, are altered in the presence of acute and chronic LBP.

## Methods/design

This protocol was developed in accordance with the Preferred Reporting Items for Systematic Reviews and Meta-Analyses Protocol (PRISMA-P) checklist (see Additional file 1) [24] and an assessment tool that evaluates the methodological quality of systematic reviews (AMSTAR) [25].

## Review question

Do people with acute and chronic LBP exhibit altered APA onset and amplitude and/or altered CPA onset and amplitude as measured by EMG, COP, and kinematics when compared to healthy individuals?

## Search strategy

The methods for this systematic review were developed using “Chapter 13” of the *Cochrane Handbook for Systematic Reviews of Interventions* [26] owing to the non-randomised nature of the studies to be included. A librarian with experience with systematic reviews was consulted for the development of the search strategy.

Databases to be included in the search are CENTRAL, MEDLINE, EMBASE, PubMed, and CINAHL. No restrictions will be placed on language. Where necessary, articles will be translated to English by independent interpreters. Keywords and medical subject headings (MeSH) related to LBP, control subjects, postural adjustments, EMG, COP, and kinematics will be used. The terms “low* back pain”, “low* backpain”, “healthy individual*”, “control*”, “healthy counterparts”, “postur* control”, “postur* balance”, “postur* adjustment*”, “postur* response*”, “centre of pressure”, “kinematics”, “electromyography*”, “muscle activity”, “neuromuscular activity”, “perturbation”, and “rapid movement” will be used in various combinations to identify relevant literature. Search strategies will be customised for each database (Additional file 2).

## Types of participants

To address the question, studies including healthy individuals and individuals with acute or chronic LBP will be eligible for inclusion. No restrictions will be placed on age, gender, or duration of symptoms to allow for the greatest number of relevant studies to be included.

## Inclusion criteria

1. Full-text studies published prior to January 2016 will be included.
2. Studies comparing the onset or amplitude of APAs and/or CPAs between people with LBP and healthy individuals, in response to voluntary movements or external perturbations measured as (a) EMG muscle activity, (b) COP, or (c) kinematic movement patterns, between healthy individuals and people with LBP.
3. Studies with levels of evidence classified as II–IV on the National Health and Medical Research Council (NHMRC) Hierarchy of Evidence for international studies [27].

## Exclusion criteria

1. Journal or conference abstract with no associated full-text article.
2. Studies that experimentally induce LBP.
3. Studies that do not include measures of onset or amplitude of APAs or CPAs, including but not limited to frequency domain analysis, linear, and non-linear measures of temporal variability.
4. Studies that include trials of animals, pregnant participants, or people with a history of spinal surgery, spinal malignancy, infection, fracture, cauda equina syndrome, metabolic or spinal inflammatory disorder.
5. Studies not comparing healthy individuals and people with LBP.
6. As APAs and CPAs are defined as muscle activity or movement occurring prior to or following a postural perturbation respectively, studies will be excluded if muscle activity or movement is not expressed relative to a perturbation.

## Outcomes

To determine differences in APA or CPA onsets, studies must report the onset of muscle activity or movement in response to a predictable perturbation (any perturbation where the participant has knowledge of the timing and magnitude of the forthcoming perturbation) or an unpredictable perturbation, respectively. For investigations of differences in APA or CPA amplitude between people with LBP and healthy individuals, studies must report the amplitude of muscle activity or movement relative to a perturbation within a defined anticipatory or compensatory epoch, respectively. Without these data, it will not be possible to determine if APA onsets, APA amplitudes, CPA onsets, or CPA amplitudes are different between healthy individuals and people with LBP. Acceptable methods for determining muscle activity include surface and fine wire EMG and ultrasonography [28, 29]. Measures of muscle activity include EMG amplitude. Measures of muscle activity via ultrasonography will only be included for analysis of muscle activity onset. Acceptable methods for determining COP include force plates and pressure plates. Measures of COP amplitude include displacement, velocity, acceleration, and reactive forces along the vertical, medio-lateral, and antero-posterior axes [30]. Kinematic measures may be recorded using various motion capture systems including optical systems with no restriction placed on type of system (i.e. active marker, passive marker), inertial systems, mechanical systems, and magnetic systems. Kinematic measures include displacement, velocity, or acceleration of the spine or pelvis and the sequence of movement of the hip or pelvis with respect to the other. EMG, COP, and kinematic analysis measures may be analysed as the slope, mean, minimum, peak, peak-to-peak, or integrated signal. Acceptable methods to determine the onset of EMG, COP, or kinematic variables include visual identification [31] and computational methods [28]. No restrictions will be placed on EMG, COP, or kinematic signal sampling or processing methods.

## Data management

Search results will be exported to EndNote citation management software, where duplicate articles will be automatically removed. Duplicates overlooked by EndNote will be removed manually. Two independent reviewers will screen the exported studies by title and abstract to determine their relevance. Potentially relevant articles will be retrieved as full text, and the two reviewers will assess against the eligibility criteria. Where the two reviewers are uncertain or cannot agree on the eligibility of individual studies, a third reviewer will act as an arbiter. Excluded studies and reasons for exclusion will be recorded.

## Data extraction

Participant characteristics, sample sizes, muscle activity, COP, kinematic measures, methods of analysis, and perturbation characteristics will be extracted and recorded on a data extraction form by two independent reviewers. The level of agreement between the two reviewers will be assessed by piloting the data extraction sheet on 10 studies that are not included in the systematic review. Agreement will be analysed by calculating a Kappa statistic (*k*) [32], and a data extraction sheet will be reconsidered if *k* is less than 0.6. Any disagreements between the reviewers during data extraction of the studies included in the systematic review will be resolved by a third reviewer. If data are missing, authors will be contacted a maximum of three times; if there is no response, the data will be considered irretrievable.

## Assessment of methodological quality

Articles that meet the eligibility criteria will be assessed independently by two reviewers. Agreement between reviewers’ assessment of methodological quality will be assessed and analysed in the same manner as the data extraction sheet. If any differences in scores arise during the systematic review, a third reviewer will act as an arbiter.

Assessment of methodological quality will occur in two levels. First, the NHMRC Hierarchy of Evidence will be used to categorise the levels of evidence of included studies [27]. This allows the identification of potential risk of bias owing to the study design. Secondly, the methodological quality of individual studies will be assessed to identify the potential extent of bias in the results. The McMaster Critical Review Form for Quantitative Studies (Additional file 3) will be used to assess the individual studies as it is applicable to all types of quantitative study designs [33, 34]. Reviewers will be required to answer 16 close-ended questions within this tool, with each item being scored as 1 for completely fulfilling the criteria and 0 for the criteria not being or being partially fulfilled. Items are then summed for a possible maximum score of 16, with 16 indicating excellent methodological quality. Total scores are divided into five categories: poor (score ≤8), fair (score = 9–10), good (score = 11–12), very good (score = 13–14), and excellent (score = 15–16), based on ranges used in previous studies [35, 36].

## Strategy for data synthesis

Differences between people with acute or chronic LBP and healthy individuals will be calculated as between-group standardised mean differences (and 95 % confidence intervals) for controlled studies and within-group standardised mean differences (and 95 % confidence intervals) for case series articles. For the purpose of the review, acute LBP will be defined as symptom duration <3 months and chronic LBP will be defined as symptom duration ≥3 months. Standardised mean differences and 95 % confidence intervals will be calculated using RevMan software (v. 5.3. the Cochrane Collaboration). Effect estimates will be interpreted according to Cohen (small ≤0.2, medium = 0.5, and large ≥0.8) [37].

An inverse-variance random-effects model will be used to pool data for each outcome measure. The *x*^2^ test will be used to detect significant heterogeneity, while the *I*^2^ test will be performed to quantify the amount of heterogeneity. Statistically significant heterogeneity will be considered present if *x*^2^*P* < 0.10. Substantial heterogeneity will be considered if *I*^2^ > 50 % [26]. If substantial heterogeneity exists, subgroup analysis will be performed to highlight potential causes for the heterogeneity. If substantial heterogeneity exists following subgroup analysis, results will be synthesised narratively and interpreted in terms of methodological rigour.

## Analysis of subgroups

If substantial heterogeneity exists following the initial meta-analysis, subgroup analysis will be performed to identity areas of methodological and clinical diversity that may account for heterogeneity. Subgrouping will be performed according to the individual’s age (greater or less than 65 years), pain intensity at time of testing (pain free or experiencing pain), and outcome measure (EMG, COP, or kinematics). Additional analysis will also be performed with poor-quality studies removed from analysis to determine if methodological quality significantly influences the results.

## Discussion

To our knowledge, this will be the first systematic review to explore if people with LBP exhibit altered APA onset and amplitude and/or CPA onset and amplitude as measured by EMG, COP, and kinematics compared with healthy individuals. This review has the potential to identify biological factors that may play a role in the persistence of LBP and areas of development based on methodological issues and gaps in the current literature. The review will aid future research that aims to identify CNS changes in the presence of LBP and mechanisms of action for LBP treatments.

## Limitations

As only full-text articles will be included, bias may be introduced by the exclusion of grey literature. This ‘publication bias’ may inflate between- and within-group differences as studies with significant or desirable results are more than likely to be published [26]. Additionally, studies will be included in the CPA amplitude analysis based on activity or movement following a perturbation and without a defined endpoint. While this may allow the inclusion of more possibly relevant studies, this type of analysis does not allow for differentiation of changes in early or late CPAs [7].

## Review status

The reviewers have commenced searching relevant studies on the electronic databases. The review is expected to be complete by April 2016.

## Additional files

Additional file 1: PRISMA-P (Preferred Reporting Items for Systematic Reviews and Meta-Analyses Protocols) checklist. Recommended items to address in a systematic review protocol. Additional file 2: Search strategies. Individual search strategies for each database intended for the systematic review. Additional file 3: McMaster Critical Review Form for Quantitative Studies.

## Abbreviations

AMSTAR: a measurement tool for the assessment of systematic reviews
APAs: anticipatory postural adjustments
CNS: central nervous system
COP: centre of pressure
CPAs: compensatory postural adjustments
EMG: electromyography
LBP: low back pain
NHMRC: National Health and Medical Research Council
PRISMA: Preferred Reporting Items for Systematic Reviews and Meta-Analyses
