# Supplementary material for: Anticipatory and compensatory postural adjustments in people with low back pain: a protocol for a systematic review and meta-analysis
Source: Syst Rev. 2016 Apr 16;5:62. doi: 10.1186/s13643-016-0242-4 (PMC4833897; doi:10.1186/s13643-016-0242-4)
Supplement: Additional file 2: — Search strategies. Individual search strategies for each database intended for the systematic review. [file 13643_2016_242_MOESM2_ESM.docx]

**Additional file 2. Search strategies.**

Searches will be limited to full text, published up until January 2016.

**CENTRAL**

ID Search

#1 kinematics

#2 MeSH descriptor: [Biomechanical Phenomena] explode all trees

#3 centre of pressure

#4 postur* response*

#5 postur* adjustment*

#6 postur* control

#7 MeSH descriptor: [Postural Balance] explode all trees

#8 control

#9 healthy counterpart

#10 MeSH descriptor: [Low Back Pain] explode all trees

#11 low back pain

#12 MeSH descriptor: [Healthy Volunteers] explode all trees

#13 MeSH descriptor: [Electromyography] explode all trees

#14 electromyograph*

#15 muscle activity

#16 neuromuscular activity

#17 perturbation

#18 movement

#19 #10 or #11

#20 #8 or #9 or #12

#21 #4 or #5 or #6 or #7

#22 #1 or #2 or #3 or #13 or #14 or #15 or #16

#23 #17 or #18

#24 #22 or #21

#25 #19 and #20 and #21 and #22 and #23

#26 #19 and #20 and #21 and #22

#27 #19 and #20 and #24

#28 #19 and #24

**MEDLINE & EMBASE**

1. exp Low Back Pain/ or low* back pain.mp.

2. low* backpain.mp.

3. healthy individual*.mp.

4. healthy counterpart*.mp. [mp=title, abstract, original title, name of substance word, subject heading word, keyword heading word, protocol supplementary concept word, rare disease supplementary concept word, unique identifier]

5. control*.mp. [mp=title, abstract, original title, name of substance word, subject heading word, keyword heading word, protocol supplementary concept word, rare disease supplementary concept word, unique identifier]

6. postur* control.mp. [mp=title, abstract, original title, name of substance word, subject heading word, keyword heading word, protocol supplementary concept word, rare disease supplementary concept word, unique identifier]

7. exp Postural Balance/ or postur* balance.mp.

8. postur* adjustment*.mp. [mp=title, abstract, original title, name of substance word, subject heading word, keyword heading word, protocol supplementary concept word, rare disease supplementary concept word, unique identifier]

9. postur* response*.mp. [mp=title, abstract, original title, name of substance word, subject heading word, keyword heading word, protocol supplementary concept word, rare disease supplementary concept word, unique identifier]

10. centre of pressure.mp.

11. kinematics.mp. or exp Biomechanical Phenomena/

12. exp Electromyography/ or electromyograph*.mp.

13. muscle activity.mp. [mp=title, abstract, original title, name of substance word, subject heading word, keyword heading word, protocol supplementary concept word, rare disease supplementary concept word, unique identifier]

14. neuromuscular activity.mp. [mp=title, abstract, original title, name of substance word, subject heading word, keyword heading word, protocol supplementary concept word, rare disease supplementary concept word, unique identifier]

15. perturbation.mp. [mp=title, abstract, original title, name of substance word, subject heading word, keyword heading word, protocol supplementary concept word, rare disease supplementary concept word, unique identifier]

16. rapid movement.mp. [mp=title, abstract, original title, name of substance word, subject heading word, keyword heading word, protocol supplementary concept word, rare disease supplementary concept word, unique identifier]

17. 1 or 2

18. 3 or 4 or 5

19. 6 or 7 or 8 or 9

20. 10 or 11 or 12 or 13 or 14 or 15 or 16

21. 17 and 18 and 19 and 20

**Pubmed**

(((((Low* back pain) OR Low* backpain)) AND (((healthy individual*) OR Healthy counterpart*) OR control*)) AND ((((postur* control) OR postur* balance) OR postur* adjustment*) OR postur* response*)) AND ((((((centre of pressure) OR kinematics) OR biomechanical phenomena) OR electromyograp*) OR muscle activity) OR neuromuscular activity)

**Cinahl**

| S1 | MW Low* back pain OR Low* back pain |
| --- | --- |
| S2 | Low* backpain |
| S3 | MW Healthy individual* OR Healthy individual* |
| S4 | healthy counterpart* |
| S5 | control* |
| S6 | MW postur* control OR postur* control |
| S7 | postur* balance AND postur* balance |
| S8 | postur* adjustment* |
| S9 | postur* response* |
| S10 | centre of pressure |
| S11 | MW biomechanical phenomena OR kinematics |
| S12 | MW electromyography OR electromyograph* |
| S13 | muscle activity |
| S14 | neuromuscular activity |
| S15 | perturbation |
| S16 | movement |
| S17 | S1 OR S2 |
| S18 | S3 OR S4 OR S5 |
| S19 | S6 OR S7 OR S8 OR S9 |
| S20 | S10 OR S11 OR S12 OR S13 OR S14 |
