# Supplementary material for: Anticipatory and compensatory postural adjustments in people with low back pain: a protocol for a systematic review and meta-analysis
Source: Syst Rev. 2016 Apr 16;5:62. doi: 10.1186/s13643-016-0242-4 (PMC4833897; doi:10.1186/s13643-016-0242-4)
Supplement: Additional file 3: — McMaster Critical Review Form for Quantitative Studies. [file 13643_2016_242_MOESM3_ESM.pdf]

# Critical Review Form - Quantitative Studies

© Law, M., Stewart, D., Pollock, N., Letts, L., Bosch, J., & Westmorland, M., 1998
McMaster University

**CITATION:**

| |
|---|
| |
| |
| |

| | Comments |
|---|---|
| **STUDY PURPOSE:**<br>Was the purpose stated clearly?<br>❍ Yes<br>❍ No | Outline the purpose of the study. How does the study apply to occupational therapy and/or your research question? |
| **LITERATURE:**<br>Was relevant background literature reviewed?<br>❍ Yes<br>❍ No | Describe the justification of the need for this study. |
| **DESIGN:**<br>❍ randomized (RCT)<br>❍ cohort<br>❍ single case design<br>❍ before and after<br>❍ case-control<br>❍ cross-sectional<br>❍ case study | Describe the study design. Was the design appropriate for the study question? (e.g., for knowledge level about this issue, outcomes, ethical issues, etc.)<br><br>Specify any biases that may have been operating and the direction of their influence on the results. |

**Comments**

| | |
|---|---|
| **SAMPLE:**<br>N =<br><br>Was the sample described in detail?<br>❍ Yes<br>❍ No<br><br>Was sample size justified?<br>❍ Yes<br>❍ No<br>❍ N/A | Sampling (who; characteristics; how many; how was sampling done?) If more than one group, was there similarity between the groups?<br><br>Describe ethics procedures. Was informed consent obtained? |
| **OUTCOMES:**<br><br>Were the outcome measures reliable?<br>❍ Yes<br>❍ No<br>❍ Not addressed<br><br>Were the outcome measures valid?<br>❍ Yes<br>❍ No<br>❍ Not addressed | Specify the frequency of outcome measurement (i.e., pre, post, follow-up)<br><br>Outcome areas (e.g., self-care, productivity, leisure). List measures used. |
| **INTERVENTION:**<br>Intervention was described in detail?<br>❍ Yes<br>❍ No<br>❍ Not addressed<br>Contamination was avoided?<br>❍ Yes<br>❍ No<br>❍ Not addressed<br>❍ N/A<br>Cointervention was avoided?<br>❍ Yes<br>❍ No<br>❍ Not addressed<br>❍ N/A | Provide a short description of the intervention (focus, who delivered it, how often, setting). Could the intervention be replicated in occupational therapy practice? |

| | Comments |
|---|---|
| **RESULTS:**<br>Results were reported in terms of statistical significance?<br>❍ Yes<br>❍ No<br>❍ N/A<br>❍ Not addressed<br><br>Were the analysis method(s) appropriate?<br>❍ Yes<br>❍ No<br>❍ Not addressed | What were the results? Were they statistically significant (i.e., $p < 0.05$)? If not statistically significant, was study big enough to show an important difference if it should occur? If there were multiple outcomes, was that taken into account for the statistical analysis? |
| Clinical importance was reported?<br>❍ Yes<br>❍ No<br>❍ Not addressed | What was the clinical importance of the results? Were differences between groups clinically meaningful? (if applicable) |
| Drop-outs were reported?<br>❍ Yes<br>❍ No | Did any participants drop out from the study? Why? (Were reasons given and were drop-outs handled appropriately?) |
| **CONCLUSIONS AND CLINICAL IMPLICATIONS:**<br><br>Conclusions were appropriate given study methods and results<br>❍ Yes<br>❍ No | What did the study conclude? What are the implications of these results for occupational therapy practice? What were the main limitations or biases in the study? |
